# Sleep symptoms in syndromes of frontotemporal dementia and Alzheimer’s disease: A proof-of-principle behavioural study

**DOI:** 10.1016/j.ensci.2019.100212

**Published:** 2019-11-04

**Authors:** Tara P. Sani, Rebecca L. Bond, Charles R. Marshall, Chris J.D. Hardy, Lucy L. Russell, Katrina M. Moore, Catherine F. Slattery, Ross W. Paterson, Ione O.C. Woollacott, Indra Putra Wendi, Sebastian J. Crutch, Jonathan M. Schott, Jonathan D. Rohrer, Sofia H. Eriksson, Derk-Jan Dijk, Jason D. Warren

**Affiliations:** a Dementia Research Centre, UCL Institute of Neurology, University College London, London, UK; b Neurology Department, Faculty of Medicine and Health Sciences, Atma Jaya Catholic University of Indonesia, Jakarta, Indonesia; c Wolfson Institute of Preventive Medicine, Queen Mary University of London, London, UK; d Department of Chemistry and Biochemistry, Faculty of Medicine and Health Sciences, Atma Jaya Catholic University of Indonesia, Jakarta, Indonesia; e Department of Clinical and Experiential Epilepsy, UCL Institute of Neurology, University College London, London, UK; f Surrey Sleep Research Centre, University of Surrey, UK; g Dementia Research Institute, UK

**Keywords:** Sleep, Frontotemporal dementia, Progressive aphasia, Semantic dementia, Alzheimer’s disease

## Abstract

Sleep disruption is a key clinical issue in the dementias but the sleep phenotypes of these diseases remain poorly characterised. Here we addressed this issue in a proof-of-principle study of 67 patients representing major syndromes of frontotemporal dementia (FTD) and Alzheimer’s disease (AD), in relation to 25 healthy older individuals. We collected reports on clinically-relevant sleep characteristics - time spent overnight in bed, sleep quality, excessive daytime somnolence and disruptive sleep events. Difficulty falling or staying asleep at night and excessive daytime somnolence were significantly more frequently reported for patients with both FTD and AD than healthy controls. On average, patients with FTD and AD retired earlier and patients with AD spent significantly longer in bed overnight than did healthy controls. Excessive daytime somnolence was significantly more frequent in the FTD group than the AD group; AD syndromic subgroups showed similar sleep symptom profiles while FTD subgroups showed more variable profiles. Sleep disturbance is a significant clinical issue in major FTD and AD variant syndromes and may be even more salient in FTD than AD. These preliminary findings warrant further systematic investigation with electrophysiological and neuroanatomical correlation in major proteinopathies.

## Highlights

• Sleep is a key concern in dementias but their sleep phenotypes are not well defined.
• We addressed this issue in major FTD and AD syndromes versus healthy older controls.
• We surveyed sleep duration, quality and disruptive events, and daytime somnolence.
• Sleep symptoms were frequent in FTD and AD and distinguished these diseases.
• Sleep disturbance is an important clinical issue across major FTD and AD syndromes.

## 1 Introduction

Sleep disturbance has recently emerged as a major issue in dementia [[1], [2], [3], [4]]. In Alzheimer’s disease (AD), impaired sleep has been linked to adverse clinical outcomes and disease pathophysiology [3,[5], [6], [7], [8], [9]]. Clinical sleep disruption in AD has associated electrophysiological [3,10] and neurochemical indices, including altered trafficking of CSF beta-amyloid_1-42_ (the soluble oligomeric species implicated in generating amyloid plaques), phosphorylated tau and pro-inflammatory peptides [1,3,4,11]. The core brain network targeted by AD pathology plays a key role in sleep-wake cycling [1,[12], [13], [14], [15], [16]]: sleep disturbance in AD may therefore establish a pathophysiological ‘vicious cycle’, whereby loss of normal restorative functions of sleep amplifies the neurodegenerative process within vulnerable neural circuitry [1,3,4,11,16]. However, it remains unclear to what extent this ‘hypnic’ mechanism extends to the other neurodegenerative syndromes [2]. In particular, sleep has not been studied systematically and remains poorly characterised in the frontotemporal dementias (FTD), a clinically and pathologically diverse group of diseases that collectively constitute a leading cause of younger onset dementia [17].

The disease process in FTD targets sleep circuitry in hypothalamus and basal forebrain [18,19] and patients with FTD have been reported to develop excessive somnolence as well as narcolepsy-like attacks, insomnia and other sleep-related symptoms [[20], [21], [22], [23]]. Sleep disturbance in FTD forms part of a much broader spectrum of homeostatic and related behavioural alterations, also affecting appetite and other biological drives [17,22,24]. Whereas AD may be uniquely associated with elevated orexin levels [3], FTD is associated with reduced plasma and CSF orexin that correlates with excessive daytime somnolence [20,25]. These observations suggest that AD and FTD may have distinct sleep phenotypes, and sleep disturbance is potentially a quantifiable biomarker and therapeutic target in these dementias [1,2,26]. However, any phenotypic segregation is likely to be modulated by heterogeneity within the FTD spectrum: the major syndromes of FTD have characteristic clinico-anatomical profiles (progressive socio-emotional dysfunction associated with variable frontal and temporal lobe atrophy in behavioural variant frontotemporal dementia, bvFTD; progressive language output failure with predominant peri-Sylvian atrophy in progressive nonfluent aphasia, PNFA; erosion of vocabulary and semantic memory with antero-mesial temporal lobe atrophy in semantic dementia, SD), underpinned by various histopathologies [17]. Moreover, we currently lack basic information about the clinical phenomenology of sleep in FTD versus AD syndromes [3,4,8,[27], [28], [29]].

Our key objectives in this proof-of-principle study were to gather initial data on the sleep symptoms experienced by patients representing canonical syndromes of FTD and AD, relative to healthy older individuals; and to compare sleep symptom profiles in these different dementia syndromes. We intended the study to provide a preliminary survey or ‘snapshot’ of sleep phenotypes across this diverse disease spectrum. We surveyed the prevalence of symptoms covering broad domains of sleep-related function that we anticipated would sample the clinical spectrum of sleep disruption and its impact on quality of life and care, based on reports by patients’ primary caregivers: these domains comprised time spent in bed overnight (as a proxy for sleep duration), subjective sleep quality, daytime impact (excessive somnolence) and associated behavioural phenomena (disruptive sleep events; a potential marker of REM behaviour disorder).

We hypothesised that sleep symptoms would be more frequent in patients with FTD and AD than healthy older individuals and would be more prevalent with advancing disease. Based on available clinical and neuroanatomical evidence, we further hypothesised that sleep symptom profiles would vary between diseases and syndromes: we predicted different sleep profiles in AD versus FTD (with relatively more prominent daytime somnolence in FTD); and similar sleep profiles in variant syndromes of AD (typical amnestic AD (tAD) and its major 'visual' variant, posterior cortical atrophy (PCA)) which share a common neuroanatomical and histopathological substrate) but divergent profiles among FTD syndromes.

## 2 Materials and methods

### 2.1 Participant characteristics and inclusion/exclusion criteria

The clinical cohort comprised 40 patients with FTD (19 with bvFTD, 11 with SD, 10 with PNFA) and 39 patients with AD (27 with tAD, 12 with PCA). Patients were recruited via a specialist cognitive clinic and all fulfilled consensus criteria for the relevant diagnosis [[30], [31], [32], [33]]. In the AD group, patients with relatively young age at onset were selected, both to avoid confounding age effects in comparison to the FTD cohort and to minimise any contribution from comorbidities such as cerebrovascular disease. All participating patients had clinically mild to moderate cognitive impairment and all were still living at home with their primary caregivers at the time of the study. As a condition of entry, every participating patient had a bed partner (the primary caregiver) who was able to provide reliable information about their nocturnal sleep and associated daytime symptoms. Twenty-five healthy older individuals from the Dementia Research Centre database with no history of significant neurological or psychiatric illness also participated; none of these healthy controls was the spouse or caregiver of a patient with dementia, in order to avoid confounding their responses by proximity to another person with dementia-associated sleep impairment. No participant had a previous (premorbid) history of obstructive sleep apnoea or other parasomnia; no male participant had a history of significant prostatism or other urological illness (to avoid confounding from comorbid nocturia).

All patients had cognitive and volumetric brain MRI findings in keeping with the syndromic diagnosis and without significant cerebrovascular burden. Twenty-four patients in the AD group (15 typical AD, 9 PCA) had undergone diagnostic lumbar puncture; each of these had a CSF total tau: beta amyloid1-42 ratio >1, consistent with underlying AD pathology. Blood samples for all patients were screened for pathogenic genetic mutations with targeted next-generation gene sequencing using the MRC dementia gene panel [34] and (to detect *C9orf72* mutations) repeat-primed PCR with expansion size derived on Southern blot; nine patients in the FTD group were found to have pathogenic mutations (three *GRN*, two *MAPT*, four *C9orf72*). Clinical and demographic characteristics of the participant groups are summarised in Table 1. Further details about genetic cases are in Table 2; all fulfilled relevant syndromic diagnostic criteria [30,31].

**Table 1:** General demographic, clinical and sleep symptom profiles of all participant groups.

General characteristic	Controls	*bvFTD*	*SD*	*PNFA*	FTD (all)	*tAD*	*PCA*	AD (all)
No. (m:f)	25 (10:15)	*19 (13:6)*	*11 (8:3)*	*10 (4:6)*	40 (25:15)	*27 (14:13)*	*12 (2:10)*	39 (16:23)
Age (years)	67.3 (7.5)	*65.3 (7.9)*	*62.0 (6.3)*	*69.9 (8.9)*	65.5 (8.1)	*64.4 (7.9)*	*62.7 (4.6)*	63.9 (7.0)
Years since symptom onset	N/A	*5.1 (4.0)*	*5.5 (2.3)*	*4.3 (2.2)*	5.0 (3.2)	*4.9 (2.7)*	*5.5 (3.3)*	5.1 (2.9)
MMSE	29.5 (0.7)	***21.0 (6.0)***	***23.1 (7.9)***	***21.8 (8.7)***	**23.2 (7.1)**	***19.7 (4.8)***	***24.0 (3.7)***	**21.0 (4.9)**
Medication use: (no.)								
AChEI	0	*1*	*0*	*1*	2	*25*	*11*	36
Memantine	0	*0*	*0*	*0*	0	*4*	*2*	6
Antidepressants†	0	*8*	*7*	*4*	19	*7*	*5*	12
Benzodiazepines	0	*0*	*1*	*0*	1	*0*	*0*	0

**Sleep symptoms†**								
Time overnight in bed (hours)	7.78 (1.36)	*8.2 (2.0)*	*9.1 (1.6)*	*8.4 (1.4)*	8.5 (1.7)*	*9.4 (1.6)*	*9.6 (1.1)*	9.5 (1.5)*
Usual time of retiring^§^	23.4 (0.75)	*22.97 (1.43)*	*22.32 (1.13)*	*22.2 (1.09)*	**22.6 (1.29)**	*22.58 (0.96)*	*22.08 (0.93)*	**22.43 (0.97)**
Usual time of rising	7.19 (1.35)	*7.05 (2.25)*	*7.46 (1.95)*	*6.60 (1.22)*	7.05 (1.94)	*7.95 (1.38)*	*7.71 (0.62)*	7.88 (1.20)
Difficulty sleeping: no. (%)	7 (28)	***16 (84)***	*7 (64)*	*6 (60)*	**29 (73)**	***15 (56)***	***8 (67)***	**23 (59)**
Daytime somnolence: no. (%)	3 (12)	***15 (79)***	***8 (73)***	***9 (90)***	32 (80)*	***14 (52)***	***6 (50)***	20 (51)*
Disruptive sleep events: no. (%)	5 (20)	*8 (42)*	*2 (18)*	*1 (10)*	11 (28)*	*2 (7)*	*1 (8)*	3 (8)*

Mean (standard deviation) values are presented unless otherwise indicated. Data for syndromic subgroups within larger disease groups (all cases of FTD and AD) are in italics. Bold indicates significant differences between patient group and healthy controls (p < 0.05); †for patients, based on caregiver reports; *significantly different between FTD and AD group (p < 0.05); †all selective serotonin reuptake inhibitors; ^§^24 hour clock times. AchEI, acetylcholinesterase inhibitors; AD, Alzheimer’s disease; bvFTD, patient subgroup with behavioural variant frontotemporal dementia; Controls, healthy control group; FTD, frontotemporal dementia; MMSE, Mini-Mental State Examination score; N/A, not applicable; no., number; PCA, patient subgroup with posterior cortical atrophy; PNFA, patient subgroup with progressive nonfluent aphasia; SD, patient subgroup with semantic dementia; tAD, patient subgroup with clinically typical Alzheimer’s disease.

**Table 2:** Sleep symptoms in patients with frontotemporal dementia and genetic mutations.

Age (decade)	Sx (yrs)	Syndrome	Gene	Sleep symptoms			
				Time overnight in bed (mean hours)	Difficulty sleeping	Daytime somnolence	Disruptive sleep events
7th	10	bvFTD	*GRN*	7.5	+	–	–
7th	1	bvFTD	*GRN*	6.5	+	+	–
7th	1	PNFA	*GRN*	9.5	+	+	–
7th	10	bvFTD	*MAPT*	9.5	+	+	–
6th	6	SD	*MAPT*	8.0	+	–	–
7th	9	bvFTD	*C9orf72*	4.0	+	+	+
8th	11	bvFTD	*C9orf72*	8.5	+	+	+
7th	5	bvFTD	*C9orf72*	10.5	+	–	+
7th	6	bvFTD	*C9orf72*	6.5	+	+	+

+, sleep symptom present; -, sleep symptom absent; *C9orf72*, mutation in chromosome 9 open reading frame 72; bvFTD, behavioural variant frontotemporal dementia; *GRN*, mutation in progranulin gene; *MAPT*, mutation in microtubule-associated protein tau gene; PNFA, progressive nonfluent aphasia; SD, semantic dementia; Sx, symptoms (duration in years).

All participants gave written informed consent. Ethical approval for the study was granted by the National Hospital for Neurology and Neurosurgery and the University College London Research Ethics Committees, in accordance with the Declaration of Helsinki.

### 2.2 Assessment of sleep-related symptoms

We adapted items from relevant domains of the Cambridge Behavioural Inventory (Revised) [35] and Pittsburgh Sleep Quality Index [36] or created these de novo, in order to capture sleep symptoms predicted to be most relevant to both FTD and AD [[1], [2], [3], [4],[20], [21], [22], [23]]. The sleep symptom survey is presented in Table 3. The survey was completed by patients’ primary caregivers (bed partners) and by healthy controls themselves. We assessed: i) estimated average total time spent overnight in bed (calculated from usual times of retiring to bed in the evening and rising from bed the following morning); ii) whether participants had experienced substantial difficulty sleeping at night (e.g., delay falling asleep, frequent arousals); iii) whether participants experienced excessive daytime somnolence (assessed by a history of taking naps or falling asleep easily and involuntarily, e.g. while watching television); and iv) whether participants had frequent disruptive sleep events (for patients, based on caregiver descriptions of nocturnal behaviours such as shouting or otherwise acting out dream content; for healthy controls, reported frequency of disturbing dream content).

**Table 3:** Customised survey to assess sleep symptoms in patients with frontotemporal dementia and Alzheimer’s disease.

Sleep-related feature	Questionnaire item
Usual time of retiring	What time does s/he / do you usually go to bed in the evening?
Usual time of rising	What time does s/he / do you usually rise from bed in the evening?
Average total time spent overnight in bed	[calculated]
Difficulty sleeping at night*	Does s/he / do you often have a delay falling asleep or frequent awakenings during the night?
Excessive daytime somnolence†	Is s/he / are you often sleepy during the day (e.g., do you take naps or fall asleep easily and involuntarily, as while watching television or eating meals)
Disruptive sleep events	[caregivers] Is s/he often restless in bed, shouting or seeming to act out dreams? [healthy controls] Do you often experience disturbing dreams (e.g., nightmares that may cause you to wake)

As a condition of entering the study, every participating patient had a bed partner (the primary caregiver) who was able to provide reliable information about their nocturnal sleep and associated daytime symptoms. Healthy controls completed the questionnaire in respect of their own sleep, commenting on the salience of disturbing dreams under the item ‘disruptive sleep events’; none of the participating healthy controls was the spouse or caregiver of a patient with dementia, in order to avoid confounding their responses by proximity to another person with dementia-associated sleep impairment. *item adapted from Cambridge Behavioural Inventory (Revised) †item adapted from Pittsburgh Sleep Quality Index.

Although various quantitative measures (e.g., based on symptom severity and/or frequency) could potentially be used in scoring individual items, for this first study we elected to record the prevalence of sleep symptoms using a straightforward, very general procedure that could be applied uniformly across items and participant groups, with minimal prior assumptions about the underlying pathophysiological processes. Accordingly, total time spent each night in bed and usual times of retiring and rising separately (on a 24 -h clock) were recorded and entered into analyses as numerical values (hours); other items were scored as present or absent.

### 2.3 Analyses

Demographic, clinical and sleep data were analysed using SPSS24®. In analyses of symptom data, multiple linear or logistic regression models were constructed on dependent variables of interest incorporating diagnostic group, age and gender as covariates. Participant group demographic and clinical data were compared using one-way analysis of variance (ANOVA) with Bonferroni post-hoc tests where indicated, independent t-tests, or chi-square tests. Data on retiring and arising times as well as time spent overnight in bed were analysed using ANOVA with Bonferroni post-hoc tests. Categorical sleep symptom data were compared between groups using 95% confidence intervals (CI) on odds ratios (OR); bias-corrected-and-accelerated bootstrap analyses with 1000 iterations were used where normality assumptions were violated (e.g., due to highly skewed data distributions). We assessed both combined disease groups (FTD and AD) and syndromic subgroups separately within each large disease group. Correlations between sleep symptoms and clinical disease (symptom) duration were assessed within the FTD and AD groups using logistic regression, covarying for age, gender and syndromic diagnosis.

A statistical threshold p < 0.05 was accepted as the significance criterion for all tests.

## 3 Results

Group comparisons for demographic, clinical and sleep symptom characteristics are presented in Table 1.

### 3.1 General participant characteristics

Participant groups did not differ in mean age (F(2, 101) = 1.591, p = 0.209), or gender distribution (χ²(2) = 4.72, p = 0.094). ANOVA revealed a significant effect of disease diagnosis on MMSE score [F(2, 101) = 19.67, p < 0.001]; Bonferroni post-hoc tests revealed no significant difference between mean MMSE scores in the combined FTD group versus the combined AD group, while both disease groups differed significantly from healthy controls. Symptom duration did not differ between the FTD and AD groups (p = 0.940). As anticipated, most (36/39) patients with AD but only two patients with FTD were taking an acetylcholinesterase inhibitor; frequency of antidepressant use did not differ significantly between the AD and FTD groups (χ²(1) = 2.32, p = 0.165), while only one patient in the study was prescribed a benzodiazepine and none was prescribed a neuroleptic medication.

### 3.2 Time overnight in bed

**Fig. 1:**
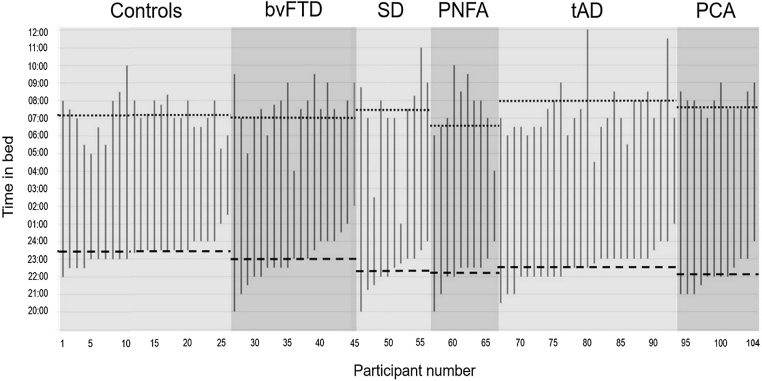
Usual daily rest periods for individual participants. Estimated usual times of retiring and rising each day (24 h clock time on the y-axis) and intervening periods of time in bed have been plotted for all individuals in each of the participant groups: healthy older controls, patients with behavioural variant frontotemporal dementia (bvFTD), semantic dementia (SD), progressive nonfluent aphasia (PNFA), typical amnestic Alzheimer’s disease (tAD) and posterior cortical atrophy (PCA). Individual plots have been ordered within groups according to estimated usual time of retiring. Average times of retiring (horizontal dashed line) and rising (horizontal dotted line) are indicated for each group.

Usual daily rest periods for all individual participants are plotted in Fig. 1 and group data for usual times of retiring and rising and time spent overnight in bed are presented in Table 1.

Time spent overnight in bed differed significantly among the FTD, AD and healthy control groups (F(2, 101) = 9.80, p < 0.001); Bonferroni post-hoc tests revealed that the combined AD group spent significantly longer on average overnight in bed than healthy controls (mean difference 1.69 h, p < 0.001); whereas the combined FTD group did not differ significantly from healthy controls (p = 0.240). Comparing disease groups, the combined AD group spent on average significantly longer overnight in bed than the combined FTD group (mean difference 0.99 h, p = 0.014). When syndromic subgroups were compared separately to healthy controls, no significant differences in average time spent overnight in bed were identified.

The usual time of retiring was on average significantly earlier in patients with FTD (mean difference 0.81 h, p = 0.011) and AD (mean difference 0.98 h, p = 0.002) than in healthy controls. These groups did not differ significantly in usual time of rising (F(2, 101) = 3.08, p = 0.054). No significant syndromic group differences in usual times of retiring or rising were identified.

The lack of syndromic differences on sleep time measures may in part reflect the wide variation in bed periods in the patient groups. Inspection of the individual data (Fig. 1) suggests that patients were more likely than healthy older controls to spend extremely long or short periods of time in bed overnight; for example, individual patients with bvFTD and tAD spent over 13 h on average in bed and average estimated time spent in bed overnight for patients with SD ranged from two to over 12 h (compared with four to 11 h for healthy controls). In addition, patients with both FTD and AD syndromes were more likely to retire early than healthy older controls (15% of the healthy control group usually retired before 11 pm compared with 47% of the bvFTD group, 64% of the SD group, 80% of the PNFA group, 56% of the tAD group and 75% of the PCA group).

### 3.3 Difficulty sleeping

Compared to healthy controls, both the combined FTD and combined AD disease groups showed significantly increased odds of experiencing difficulty sleeping (for FTD, OR 9.25, p = 0.001, CI [0.94, 4.14]; for AD, OR 5.71, p = 0.008, CI [0.39, 3.41]). The combined FTD and AD groups did not differ significantly in their odds of experiencing difficulty sleeping (OR 1.88, p = 0.225, CI [-0.5, 1.84]).

Compared to healthy controls, the FTD and AD syndromic subgroups showed variably increased odds of experiencing difficulty sleeping: this was most pronounced in the bvFTD subgroup (OR 16.71, p = 0.001, CI [1.44, 22.75]) while the SD and PNFA subgroups did not show a difference in odds compared to controls (for SD, OR 4.73, p = 0.07, CI [-0.31, 5.31]; for PNFA, OR 6.68, p = 0.05, CI [-0.1, 50.80]). Both AD syndromic groups showed a significant effect (for tAD, OR 4.79, p = 0.032, CI [0.07, 3.71]; for PCA, OR = 6.98, p = 0.017, CI [0.48,5.12]). Around 80% of patients with bvFTD and around 60% of patients in other syndromic groups (compared with 27% of healthy older controls) experienced some difficulty sleeping (Table 1).

### 3.4 Excessive daytime somnolence

Relative to healthy controls, both the combined FTD and AD disease groups and all syndromic subgroups showed significantly increased daytime somnolence (for FTD, OR 23.01, p = 0.001, CI [2.20, 23.01]; for AD, OR 8.96, p = 0.005, CI [0.84, 21.66]). The combined FTD group was significantly more likely to have excessive daytime somnolence than the combined AD group (OR 3.69, p = 0.014, CI [0.34, 2.71]). Around 80% of patients with FTD syndromes and 50% of patients with AD syndromes experienced excessive daytime somnolence (Table 1).

### 3.5 Disruptive sleep events

The combined FTD and AD disease groups and syndromic subgroups did not differ significantly from healthy controls overall in their propensity to experience disruptive events associated with sleep. The combined FTD group showed higher odds of experiencing disruptive sleep events than the combined AD group (OR 4.3, p = 0.042, CI [0.09, 20.2].

### 3.6 Genetic mutation cases

Of the nine FTD patients with known mutations in the *GRN, MAPT* or *C9orf72* genes (see Table 2), all experienced difficulty sleeping and six (representing all mutations) experienced excessive daytime somnolence. All patients with *C9orf72* mutations experienced disruptive sleep events, while this was not reported for patients with other mutations.

### 3.7 Sleep symptom correlates

Over the combined patient cohort, difficulty sleeping and excessive daytime somnolence were strongly associated (p < 0.001); there were no other significant correlations among sleep symptoms. Overall clinical disease (symptom) duration was not significantly correlated with the presence of sleep symptoms in either the FTD group or the AD group.

## 4 Discussion

Here we have shown that sleep disturbance is a substantial clinical issue in diverse dementia syndromes representing the major phenotypes of FTD and AD. Difficulty falling or staying sleep at night and excessive daytime somnolence were significantly more frequently reported for patients with both FTD and AD than healthy age-matched individuals, occurring in over half of the AD group and around three-quarters of the FTD group. Patients with FTD were more likely to experience disruptive sleep events than patients with AD. Patients with FTD and AD habitually retired earlier and AD spent on average significantly longer in bed overnight than did healthy older individuals. Excessive daytime somnolence was significantly more frequent in the FTD group than the AD group overall. Our findings are comparable to estimates of the overall frequency of sleep disturbance in previous AD and FTD case series [20,22,[37], [38], [39]], corroborating previous suggestions that sleep alterations may be even more salient in FTD than AD. Information about particular syndromic profiles of sleep disturbance within the broad disease groupings of FTD and AD remains very limited; ours is the first study to assess sleep symptoms systematically across the major syndromes of FTD and AD. Differentiation of syndromes within the broad FTD and AD disease groupings here was limited, likely reflecting both the relatively small sample size for particular syndromic groups and wide individual variation among patients. However, our data support previous evidence that bvFTD tends to be associated with poor sleep, potentially reflecting diverse effects on sleep quantity, quality and timing in individual patients across the FTD spectrum [21,[40], [41], [42]].

While we do not have direct neuroanatomical or histopathological correlation in this study, our findings are in line with the known pathological anatomy of these neurodegenerative disorders. Both FTD and AD are associated with pathological involvement of circadian and sleep regulatory networks traversing hypothalamus, basal forebrain and mesial temporal lobe and associated disruption of homeostatic drives and mechanisms [1,2,[17], [18], [19],^22^,^24^,[43], [44], [45]]. Disruption of this circuitry might affect habitual times of retiring and rising, as well as the stability of sleep and wake phases [40,41,46]. In healthy individuals, consolidation of the circadian sleep-wake cycle is achieved by a coupling of reciprocal drives to wakefulness and sleep; this coupling maintains daytime wakefulness and preserves night-time sleep, opposed by increasing pressure from the reciprocal circadian drive [47,48]. Dysfunction of circadian control circuitry would disrupt the balance of these reciprocal drives, leading to fragmentation of both sleep and wakefulness [49,50]. This putative circadian dysrhythmia may aggravate the impact of accumulating wakefulness on an already vulnerable brain. One behavioural consequence might be earlier bedtimes. However, in addition to altered sleep-wake functioning *per se*, circadian behaviour is likely to be influenced by the subjective distress attending a given ‘objective’ level of sleep disturbance, how this is modulated by social context and how it is communicated by patients to their caregivers. Both bvFTD and SD may lead to altered sensitivity and abnormal behavioural responses to homeostatic derangements [[43], [44], [45]]. All three major FTD syndromes have candidate neuroanatomical substrates for abnormal circadian regulation and altered behavioural responses to sleep disruption in vulnerable fronto-subcortical networks [17,51]: we propose that involvement of these networks may account for the excessive daytime somnolence reported here in patients with PNFA as well as contributing to the pathogenesis of this symptom in bvFTD and SD. The extent to which sleep habits in FTD syndromes may reflect pathological behaviours per se, disengaged from homeostatic mechanisms, remains unresolved.

The present findings hint that sleep syndromes may have histopathological or genetic associations. The similarity of the sleep symptom profiles exhibited here by patients with tAD (led clinically by memory decline) and its major syndromic variant PCA (led clinically by visuo-spatial impairment) are in line with shared involvement of the temporo-parieto-subcortical ‘default mode network’ [52] and with other evidence for pathophysiological convergence in these syndromes [53]. Sleep disturbance may be a hallmark of AD pathology and it remains to be established how far syndromic variation modulates the hypnic expression of this pathology. On the other hand, our data raise the possibility of distinct molecular signatures of sleep disturbance within the FTD spectrum. The uniform association of disruptive sleep events with *C9orf72* mutations (but not with other FTD mutations or AD syndromes) was a striking feature of this cohort. This observation is in line with previous reports of disordered REM behaviour in association with *C9orf72* mutations [[54], [55], [56]] and accords with the neuroanatomical signature of this genetic FTD subtype, which characteristically involves a thalamo-parietal network implicated in the generation of REM sleep [57,58]. It is clear, however, that any genetic basis for sleep symptomatology is likely to be complex: sleep symptoms were reported by all patients with FTD mutations here and severe sleep phenotypes have also been previously reported with *MAPT* mutations, in human patients and in animal models [[59], [60], [61], [62]]. With respect to the prevalence of disruptive sleep events in AD syndromes, our findings are in line with previous studies of tAD but substantially lower than the frequency of nightmares reported by Ridha and colleagues in their PCA cohort [63]. This apparent discrepancy between PCA studies may signify a shift in propensity to experience sleep disruption (including disturbing dreams) with treatment phase: the previous data [63] relate to patients starting acetylcholinesterase inhibitor treatment, whereas our data relate to patients already maintained on treatment. Cholinergic pathways are known, for example, to play a critical regulatory role in REM sleep [64]: it is plausible that upregulation of acetylcholine receptors in the face of reduced cholinergic transmission in PCA (or tAD) could lead to an initial ‘rebound’ of REM phenomena (and associated sleep disruption) following the introduction of pro-cholinergic therapy, which then settles into a new equilibrium state with ongoing cholinergic degeneration [65,66]. However, comparisons between studies should be made with caution, as we did not survey dream content specifically here, nor do we have electrophysiological data for these patient cohorts.

Our findings have important clinical implications. Sleep is a viable target for development of novel biomarkers and incorporation into clinical trials; this is a theme that has emerged strongly in AD but not, so far, in FTD. The present data provide a *prima facie* case for further systematic exploration of the 24 -hour sleep-wake cycle as a source of candidate biomarkers in FTD. Moreover, sleep disruption is a major cause of caregiver burden and therefore a key management issue across dementia syndromes [8,42,67]. This is likely to require an approach tailored to particular dementia syndromes, especially in light of the very prominent behavioural dysregulation in bvFTD and SD [17]. Along with therapeutic opportunities, this implies a need for some caution, as patients with FTD as well as AD are potentially vulnerable to iatrogenic sleep disruption.

Our study has several limitations that suggest our findings should be interpreted with some caution and which should motivate future work. The first priority is to validate our sleep symptom survey and the findings in this participant cohort prospectively in other cohorts. Administering the survey questions to a larger cohort of healthy individuals would be required to determine their sensitivity and specificity in discriminating disease effects from those of healthy ageing. Further, the present findings should be corroborated using standardised sleep assessment scales and grading of symptom frequency and severity, in larger patient cohorts and based on reports collected in parallel from patients and caregivers. Larger cohorts will be particularly important given the wide individual variation observed across and between syndromic groups within the spectrum of FTD and AD.

More fine-grained analysis of sleep symptoms and a wider representation of symptoms will be leading priorities. For example, in this study we deliberately avoided including patients with a prior history of obstructive sleep apnoea in order to avoid the potentially confounding issue of dual diagnoses but sleep disordered breathing is itself likely to constitute an important risk factor in the pathogenesis of AD [3,68]. Moreover, the sleep symptom categories surveyed here were intentionally broad and inclusive. ‘Time spent in bed’ does not necessarily equate to time asleep (indeed, increased time in bed could signify non-refreshing, interrupted sleep). ‘Difficulty sleeping’ encompasses difficulty falling asleep and difficulty staying asleep, which may have quite different pathophysiological significance [[1], [2], [3], [4]]. ‘Daytime somnolence’ encompasses napping behaviour, which in turn may be voluntary or involuntary and may have prognostic significance [69]; napping frequency is potentially a readily quantifiable biomarker. ‘Disruptive sleep events’ could have various causes, including but not limited to abnormal REM sleep behaviour. It is further possible that this symptom descriptor indexed different phenomena in patients versus healthy controls: in controls, for example, it is likely to have captured chiefly directly-experienced disturbing dream content leading to awakenings, whereas patients’ caregivers were in a position to report on various forms of sleep disruption (though could only infer dream content).

Our sleep symptom survey was based on self-reporting of sleep symptoms by healthy controls versus second-person (caregiver) reporting for patients: use of a uniform symptom survey protocol across the participant cohort will be required to ensure that similar phenomena are indeed being captured in each participant group. While patient self-reports are potentially confounded by memory and behavioural impairments, these will be required to capture subjective correlates of sleep disruption (particularly in syndromes such as bvFTD and SD that are likely to disconnect subjective awareness from objective deficits) as well as to compare patients’ experience more directly with that of healthy controls. Self-reporting by patients would facilitate ratings of symptom severity and frequency, which are problematic to achieve second-hand but ultimately necessary to provide a more detailed picture of sleep phenomenology in neurodegenerative diseases. It will also be important to relate sleep symptoms to other indices of daily life functioning and to clinical prognostic outcomes. Medication use is a further important and potentially highly relevant variable: in our cohort, both the AD and FTD groups were taking antidepressants while there was a clear disproportion in the use of acetylcholinesterase inhibitors by the AD group. This reflects widespread prescribing practice in people with dementia and thus ‘real world’ experience. However, both drug classes can potentially affect sleep quantity and quality and further work is required to differentiate pharmacological from endogenous disease effects.

A further key requirement in order to evaluate and validate the biomarker potential of any sleep symptom scale will be to assess symptoms longitudinally and to ground symptom reports in objective pathophysiological measures of circadian motor activity (actigraphy) and most importantly, changes in electrophysiological sleep architecture using polysomnographic techniques, over the entire 24 -h circadian cycle. This will also be essential for excluding subclinical and intercurrent (premorbid) sleep disorders. From a neurobiological perspective, there will be considerable value in correlating sleep phenotypes with neuroimaging modalities that can examine the structural and functional integrity of circadian networks in particular FTD and AD syndromes and with neurochemical assays that can address orexinergic and related candidate mechanisms which may drive sleep disruption in these diseases. Combining data from multiple specialist centres is a powerful tool for assessing phenotypic correlates of rare genetic disease subtypes, and potentially, for detecting sleep changes that may predate other clinical symptoms [70].

## 5 Conclusions

This first clinical comparison of patient cohorts representing canonical FTD and AD syndromes has underlined that sleep-related symptoms are a significant issue across these diseases, with some evidence for differentiation of pathologies. Our findings, though preliminary, provide proof-of-principle to justify undertaking future larger scale validation and correlative studies, in order to characterise in detail the role of sleep disturbance in promoting clinical symptoms and signalling pathophysiology in major dementias.
